# Can a supranational medicines agency restore trust after vaccine suspensions? The case of Vaxzevria

**DOI:** 10.1371/journal.pone.0277554

**Published:** 2022-11-30

**Authors:** Andrea Albanese, Francesco Fallucchi, Bertrand Verheyden

**Affiliations:** 1 Luxembourg Institute of Socio-Economic Research (LISER), Esch-sur-Alzette, Luxembourg; 2 Department of Economics, University of Bergamo, Bergamo, Italy; The University of Kansas, UNITED STATES

## Abstract

Over the first half of March 2021, the majority of European governments suspended Astrazeneca’s Vaxzevria vaccine as a precaution following media reports of rare blood clots. We analyse the impact of the European Medicines Agency’s (EMA) March 18th statement assuring the public of the safety of Vaxzevria and the immediate reinstatement of the vaccine by most countries on respondents’ intention to get vaccinated. By relying on survey data collected in Luxembourg and neighbouring areas between early March and mid-April, we observe that the willingness to be vaccinated was severely declining in the days preceding the EMA statement. We implement a regression discontinuity design exploiting the time at which respondents completed the survey and find that the vaccine reinstatement substantially restored vaccination intentions.

## 1 Introduction

In 2019, the World Health Organization (WHO) had declared vaccine hesitancy as one of the ten main threats to global health. One year later, public authorities around the world welcomed vaccines as an effective solution to alleviate pressures on health systems and on the economy. Despite months of lockdown and millions of deaths, vaccine hesitancy and antivax movements immediately challenged the vaccination campaign [1]. Concerns related to vaccines were further fueled in early March 2021, when European media reported rare cases of blood clots among people who received Astrazeneca’s Vaxzevria vaccine. This led European governments to implement a large and uncoordinated wave of precautionary suspensions. By March 15th, 18 countries had suspended Vaxzevria, pending an official statement by the European Medicines Agency (EMA), the EU drug regulator. On March 18th, the EMA made gave a press conference stating that “the vaccine’s proven efficacy in preventing hospitalization and death from COVID-19 outweighs the extremely small likelihood of developing” blood clots, and recommended the vaccine’s use. Within a few hours, 15 governments reintroduced Vaxzevria.

This case study provides a natural experiment to assess the impact of a supranational health agency’s communication on vaccine hesitancy. In this paper, we use a regression discontinuity design (RDD) to measure the impact of the EMA’s statement. We use survey data on the attitudes towards COVID-19 measures of residents of Luxembourg and neighboring regions of Belgium, France and Germany between early March and mid-April 2021. The data contains information on vaccination intentions, relevant socio-demographic characteristics and the exact time at which individuals responded to the survey. Our RDD approach uses the time of the EMA press conference as cut-off (March 18th at 17:00 CET) and exploits the information of the time of survey responses in order to measure the causal impact of the EMA’s statement. We find an immediate and statistically significant effect on respondents’ vaccination intentions, which results from the combination of the EMA’s declaration and of the ensuing coordinated reinstatement of the vaccine by the EU member states. Our results are robust to validation tests, density tests and RDD tests on covariates.

Most papers assessing the effectiveness of public interventions against vaccine hesitancy are generally focused on policies which target specific populations and involve interpersonal interactions. [2] provide a meta analysis of 33 studies showing that community-based interventions (generally targeting parents or caregivers of children via home visits or information campaigns through community health workers, as well as reminder interventions), monetary incentives aimed at alleviating financial constraints [3], and technology-based health literacy have significant effects. However, less is known about the effectiveness of large scale official communications about vaccine safety. Our paper contributes to this scarce literature, which was essentially concentrated on the case of the joint vaccine against measles, mumps and rubella (MMR). False concerns of a possible link between MMR vaccination and autism indeed led to a decline in that vaccine -as well as in other vaccines- uptake in many countries. The misinformation’s overturn, first by researchers and then by public authorities, allowed to raise vaccine uptake [4–6]. An important difference between MMR and COVID-19 is that in the latter the decision to get vaccinated concerns both children and adults themselves. This distinction is relevant, as vaccine hesitancy appears to be stronger when it concerns children. Indeed, the fact that parents may have decided to receive a COVID-19 vaccine does not prevent them from being hesitant when it comes to vaccinating their children [7]. For instance, the perceived usefulness of the vaccine may be significantly lower for children. Also, the perceived lack of adequate data and concerns of long-term side effects may be stronger when children are concerned [8]. This higher hesitancy translates into lower vaccination rates among young children than among adults [9]. As far as our paper is concerned, our variable of interest is a person’s own intention to be vaccinated on a sample exclusively composed of adults.

We first present the descriptive statistics of our sample, followed by OLS estimations on the individual determinants of the willingness to be vaccinated. We then present the RDD estimates on the effect of the EMA statement, and conclude.

## 2 Methods

We first describe the data and provide descriptive statistics. We then present regression results highlighting the role of socio-demographic characteristics, perceptions and beliefs on the intention to be vaccinated. Finally, we introduce the regression discontinuity design, explain how it applies to the context of the EMA’s statement, and present estimates of the statement’s causal impact on intentions to be vaccinated.

### 2.1 Data and descriptive statistics

Our cross-sectional data was obtained from an online survey conducted among the residents of Luxembourg and the border regions. The survey was organized by Luxembourg Institute of Socio-Economic Research (LISER) in collaboration with the University of Luxembourg and advertised at the beginning of March on social media and on local websites. This survey was reviewed and approved by the Ethics Review Panel of the University of Luxembourg. To participate in the online survey, the individuals had to tick the consent box on the data protection notice. Data were anonymised before being received by the authors, therefore no individual could be identified in this study.

After a general section on demographic characteristics, respondents were redirected to one of four randomly assigned blocks of questions covering various themes. Our block of interest, which 696 individuals completed, concerns attitudes towards COVID-19 measures. Table 1 shows the summary statistics of the survey respondents. Over the survey period (beginning of March to mid-April 2021), 83% of respondents claimed that they were willing to be vaccinated. These respondents were mostly women (67%), with a representative proportion of adults employed (78%) and with some tertiary education (55%). The sample is composed of 59% of individuals who have the Luxembourg nationality. The median household income interval is between €6,000 and €8,000, and 37% of respondents’ have a household income strictly above it. COVID-19 is considered dangerous by 68% of our sample, which contains 44% of individuals above the age of 50. Almost two thirds of the sample watch television at least once per day to get informed about the news. Finally, 64% of respondents have a strong confidence in the Luxembourgish government’s action, whereas 36% either have limited trust or no trust, or did not want to express an opinion. Following the same classification, 48% did not express strong confidence in the scientific community.

**Table 1:** Descriptive statistics of the surveyed sample.

	Proportion	S.D.
Willingness to be vaccinated	0.83	(0.38)
Woman	0.67	(0.47)
Luxembourg national	0.59	(0.49)
Single	0.14	(0.35)
Graduate	0.55	(0.50)
Employed	0.78	(0.41)
Age ≥ 50	0.43	(0.50)
Considers COVID-19 to be dangerous	0.68	(0.47)
Household income above the median	0.37	(0.48)
Daily TV information	0.62	(0.49)
Limited trust or no trust in government	0.36	(0.48)
Limited trust or trust in science	0.48	(0.50)
N	673	

### 2.2 Determinants of the willingness to be vaccinated

A vast literature analyses the determinants of the willingness to be vaccinated. Vaccine hesitancy is linked to low education and income [10], to minority ethnic groups [11], to the use of specific information channels [12, 13] as well as to personality traits [14, 15]. Table 2 provides estimates of a linear probability model with incremental sets of covariates. Results from these regressions are in line with previous research on the determinants of vaccination propensity, as the willingness to be vaccinated does not significantly differ between women and men, or between single and married respondents. Respondents who attained higher education are significantly more willing to be vaccinated than others in all specifications. We also find a weak positive effect among employed individuals and among respondents above 50 years of age, whereas having a household income above the median plays no role on vaccination intentions. Respondents who consider COVID-19 to be dangerous given their age have a significantly stronger intention to be vaccinated, by about 20 percentage points. Getting informed through television at least once per day is associated with a higher propensity to be vaccinated. Finally, respondents who do not express a strong degree of trust in the government’s action, and/or in the scientific community, are also less willing to be vaccinated.

**Table 2:** Determinants of the willingness to be vaccinated: Linear regressions.

	(1)	(2)	(3)
Woman	0.039	0.0133	0.015
	(1.26)	(0.46)	(0.53)
Luxembourg national	0.053*	0.045	0.042
	(1.76)	(1.58)	(1.55)
Single	0.040	0.052	0.052
	(0.96)	(1.32)	(1.35)
Graduate	0.127***	0.129***	0.089***
	(4.27)	(4.50)	(3.18)
Employed	0.060	0.074**	0.075**
	(1.63)	(2.14)	(2.26)
Age ≥ 50	0.123***	0.060**	0.066**
	(3.99)	(1.99)	(2.30)
Considers COVID-19 to be dangerous		0.244***	0.202***
		(8.28)	(7.04)
Household income above the median		0.036	0.022
		(1.23)	(0.80)
Daily TV information		0.108***	0.108***
		(3.73)	(3.89)
Lack of trust in government			-0.123***
			(-4.22)
Lack of trust in science			-0.134***
			(-4.77)
Constant	0.597***	0.387***	0.548***
	(11.24)	(7.06)	(9.73)
R^2^	0.048	0.166	0.237
N	673	673	673

*t* statistics in parentheses

* *p* < 0.1,

** *p* < 0.05,

*** *p* < 0.01

### 2.3 Regression discontinuity design

On March 18th the EMA held a press conference to provide assurance about the safety of the Vaxvevria vaccine. We consider this statement –and the immediate reinstatement of the vaccine by 15 out of the 18 European governments that had suspended it– as our treatment. This treatment is thus to be interpreted as a strong multilateral signal from both medical and governmental institutions aimed at restoring trust in a period of turmoil.

The regression discontinuity design (RDD) is an identification strategy for observational studies that relies on the idea that around the cutoff a quasi-experiment was conducted. This means that around the cutoff the treatment allocation is as good as random and the individuals in the treated and control group are similar in all the relevant observable and unobservable characteristics. Our identification strategy is thus based on the comparison of the intentions to be vaccinated between individuals who responded shortly before and shortly after the announcement, on March 18th at 17:00. We therefore implement an RDD estimator using the time of response as the running variable. We follow the standard approach of the literature, running a local linear regression using the optimal mean squared error criterion for each side of the cutoff and triangular kernel weights [16].

The RDD estimator is obtained by running a local linear regression using the optimal mean squared error criterion for each side of the cutoff and triangular kernel weights [16]. This regression is based on the following linear model:

$$\begin{array}{ccc} y_{i} & = & \alpha +\delta \cdot 1\left(z_{i}\geq 17:00,18/03/21\right)+\beta z_{i}\cdot 1\left(z_{i}<17:00,18/03/21\right)\\ & + & \gamma z_{i}\cdot 1\left(z_{i}\geq 17:00,18/03/21\right)+\varepsilon_{i}, \end{array}$$ (1)

where

- *y*_*i*_ is the outcome variable, which is equal to 1 if respondent *i* intends to be vaccinated and zero otherwise;
- *α* is the constant term;
- *z*_*i*_ is the exact time at which respondent *i* finished the survey;
- 1(·) is the indicator function, which is equal to 1 if the argument is true. Therefore, $1(z_{i}\geq 17:00,18/03/21)$ is a dummy indicator equal to 1 if respondent *i* completed the survey after the EMA statement.
- *δ* is the causal effect of the EMA statement on the outcome;
- $\beta z_{i}\cdot 1\left(z_{i}<17:00,18/03/21\right)$ is the linear spline on the left of the cutoff.
- *γz*_*i*_ ⋅ 1(*z*_*i*_ ≥ 17: 00, 18/03/21) is the linear spline on the right of the cutoff;
- *ε*_*i*_ is the idiosyncratic error term (with zero conditional mean).

**Fig 1:**
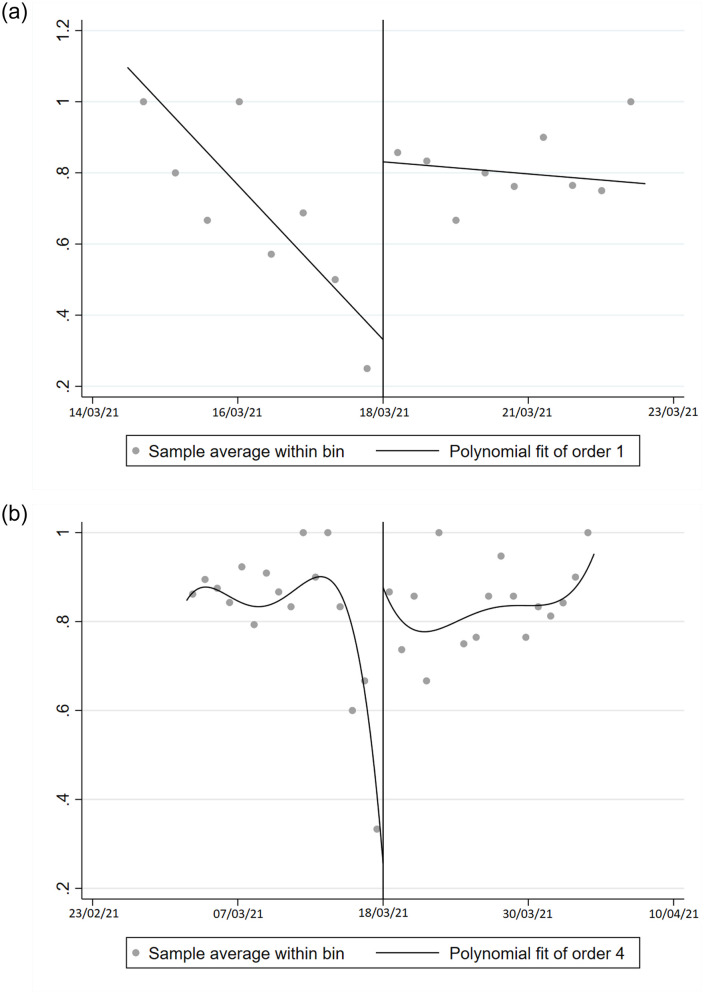
The impact of the EMA statement on the willingness to be vaccinated. *Notes*: These graphs show RDD plots for the dependent binary variable equal to 1 if the individual is willing to be vaccinated. Graph (a) is obtained using a local linear polynomial regression with triangular weights and bandwidth following the optimal mean squared error criterion in [16]. Graph (b) is obtained using a global -quartic polynomial and uniform weights.

**Fig 2:**
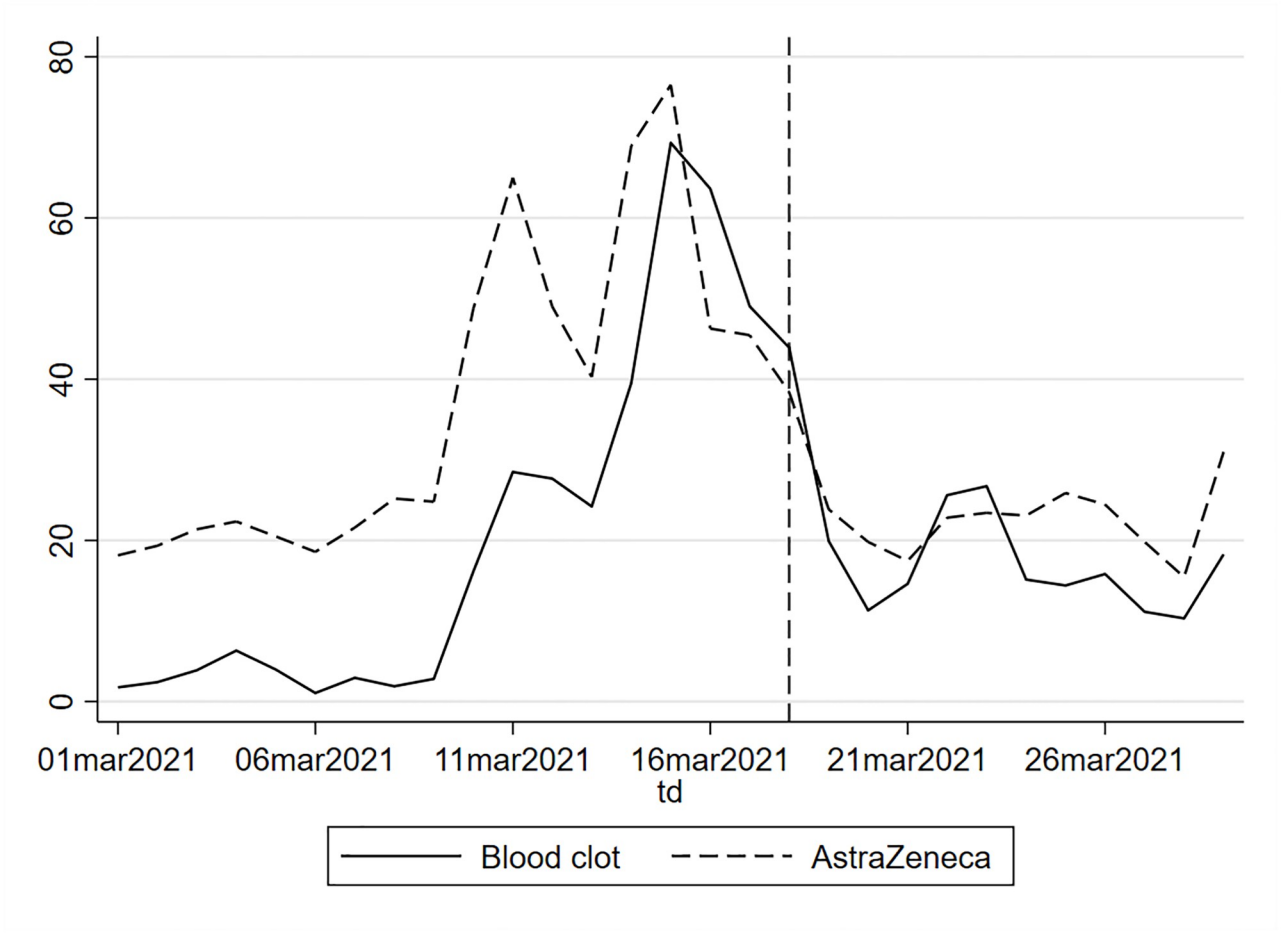
Google Trends searches for “Astrazeneca” and “blood clot” in the Greater Region of Luxembourg. The vertical dashed line indicates the day of the EMA statement. *Notes*: Google Trends index for searches for “Astrazeneca” and “blood clot” in Luxembourg, Wallonia (Belgium), Saarland and Rhineland-Palatinate (Germany) and Lorraine (France) weighted by regional population size. Moving average over two days.

We implement two extensions of this baseline model. First, we add a set of control variables *X*, which include the determinants used in Section 2.2 as well as the time of responses (morning, afternoon, evening/night) and the day of the week (weekday or weekend). Time-of-response variables are aimed at controlling for changes in the composition of respondents over specific moments of the day and week. Second, we allow for a local quadratic spline. In Fig 1 and Table 3, we report the RDD estimates of the effect of the EMA statement of the 18th of March. Column (1) shows the estimates from the local linear regression of Eq (1) without covariates. Column (3) provides linear regression results controlling for the covariates used in the preliminary analysis and the time-of-response variables. Columns (2) and (4) follow the same logic (without and with covariates) but using a more flexible (local quadratic) spline. Fig 1 provides a graphical representation of the results obtained in columns (3) and (4), i.e. local linear regression and spline with controls. We find a substantial positive effect of the EMA statement on the intention to be vaccinated of almost 50 percentage points. This effect is statistically significant at the 1% level and robust to different specifications. Considering the overall evolution of the outcome, the EMA statement was able to sharply invert the collapsing confidence observed in our data. This decline in intentions to be vaccinated in the pre-EMA period is in line with Google Trends data, which shows an increase in searches for “blood clot” and “Astrazeneca” in the days preceding the EMA’s statement across the “Greater Region”, i.e. Luxembourg, France, Germany and Belgium (see Fig 2).

**Table 3:** RDD estimates.

	(1)	(2)	(3)	(4)
Effect at cut-off	0.484***	0.518***	0.488***	0.476***
Robust *p*-value	0.007	0.004	0.001	0.008
Robust 95% CI	[0.143; 0.926]	[0.187; 0.969]	[0.213; 0.828]	[0.133; 0.879]
Y right	0.827	0.829	0.816	0.831
Y left	0.343	0.311	0.325	0.219
BW Loc. Poly. [h]—left	14/03/21 02:42	10/03/21 01:03	14/03/21 06:46	13/03/21 20:04
BW Loc. Poly. [h]—right	24/03/21 20:37	31/03/21 16:23	24/03/21 03:59	29/03/21 23:58
BW Bias [b]—left	10/03/21 20:53	07/03/21 22:28	11/03/21 09:42	11/03/21 20:03
BW Bias [b]—right	30/03/21 13:29	04/04/21 17:42	29/03/21 04:53	01/04/21 13:04
Order loc. poly. [p]	1	2	1	2
Order bias [q]	2	3	2	3
Covariates	No	No	Yes	Yes
N	c	696	673	673
Eff. N estimate [h]	132	328	128	239
Eff. N bias [b]	280	413	244	306

*Notes*: The dependent binary variable is equal to 1 if the individual responds that they are willing to be vaccinated. *z* is the running variable on the time of survey completion, with a cut-off on the 18th of March at 17:00. We follow [16] with the following options: triangular kernel; variance–covariance matrix estimated using the heteroskedasticity-robust nearest-neighbour variance estimator. Different models: (1) local linear polynomial regression based on the MSE-optimal bandwidth selector for each side of the cut-off, (2) local quadratic polynomial, (3) local linear polynomial regression adding covariates, (4) local quadratic polynomial adding covariates. The table shows the optimal bandwidth for each side of the cut-off for the estimate (h) and the bias (b).

* significant at the 10% level,

** significant at the 5% level,

*** significant at the 1% level.

For these estimates to correctly to measure the causal effect of the EMA’s statement on vaccination intentions, the RDD relies on a key assumption of continuity of the potential outcomes. This assumption requires that in the absence of treatment, the outcome would have followed a continuous evolution. This implies that individuals are unable to manipulate the forcing variable in a systematic way around the cutoff. Otherwise, the observed difference between the two groups would include confounding factors on top of the treatment effect. In our specific setting, this means that the decision to participate in the survey at a given moment should not be affected by the EMA’s statement. If certain individuals with a specific propensity to vaccinate systematically decided to participate in the survey following (or in anticipation of) the EMA’s statement, then our results would also include this change in the respondents’ composition. Such a change is however very unlikely since (i) only one fourth of survey respondents were assigned the module on attitudes to COVID-19 measures, (ii) this assignment was random and (iii) the survey questionnaire was not public.

**Fig 3:**
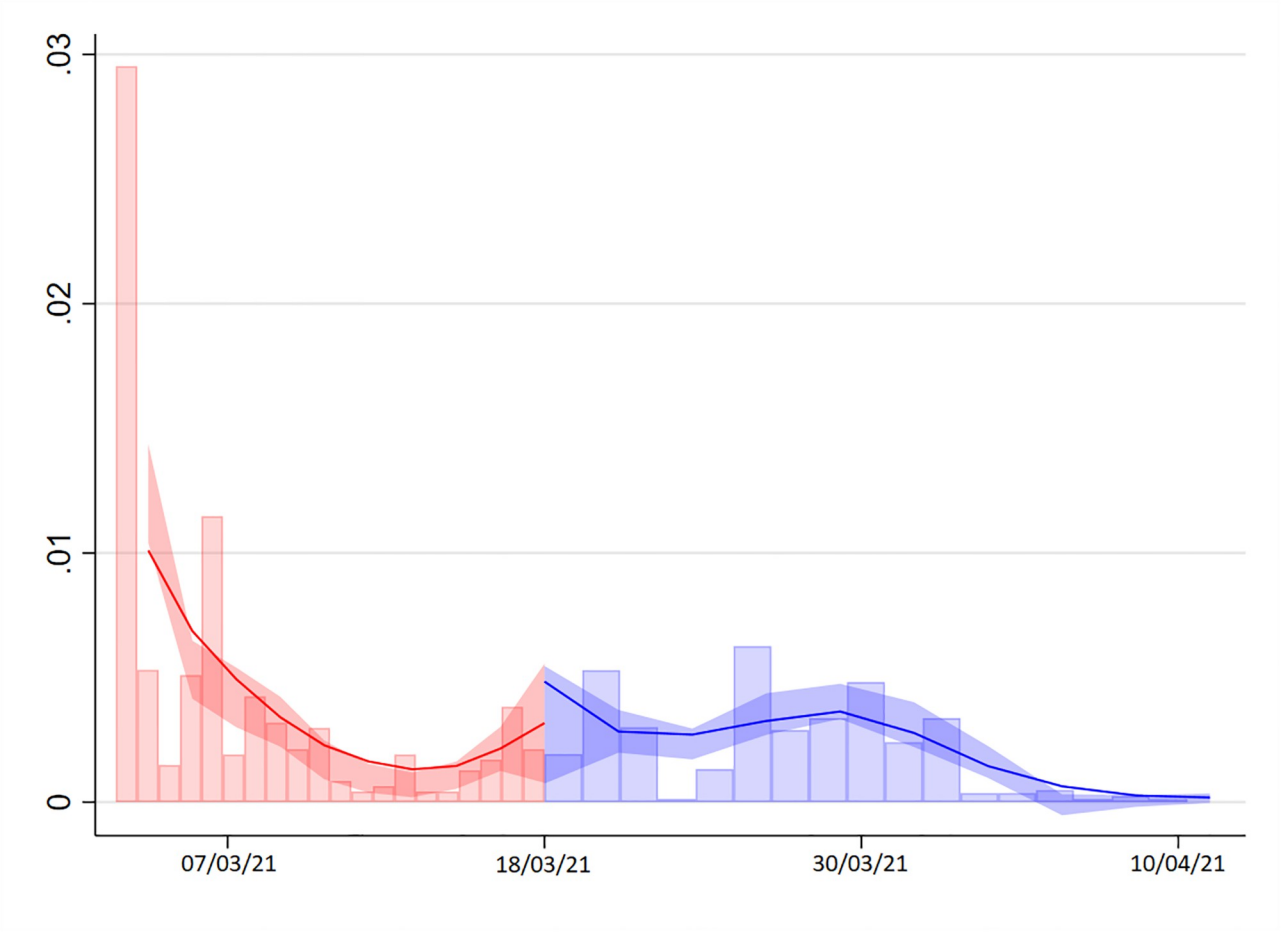
Density of survey responses over time. Notes: Manipulation test using the local polynomial density estimators proposed in [17, 18]. Stata command rddensity. A local quadratic approximation with kernel triangular weights is used to construct the density estimators, while a cubic approximation is used for the bias-corrected density estimator. The density estimation method is unrestricted (two-sample). Robust bias-corrected statistic with jackknife standard errors and uniform confidence interval at 95% level (2000 of simulations).

**Fig 4:**
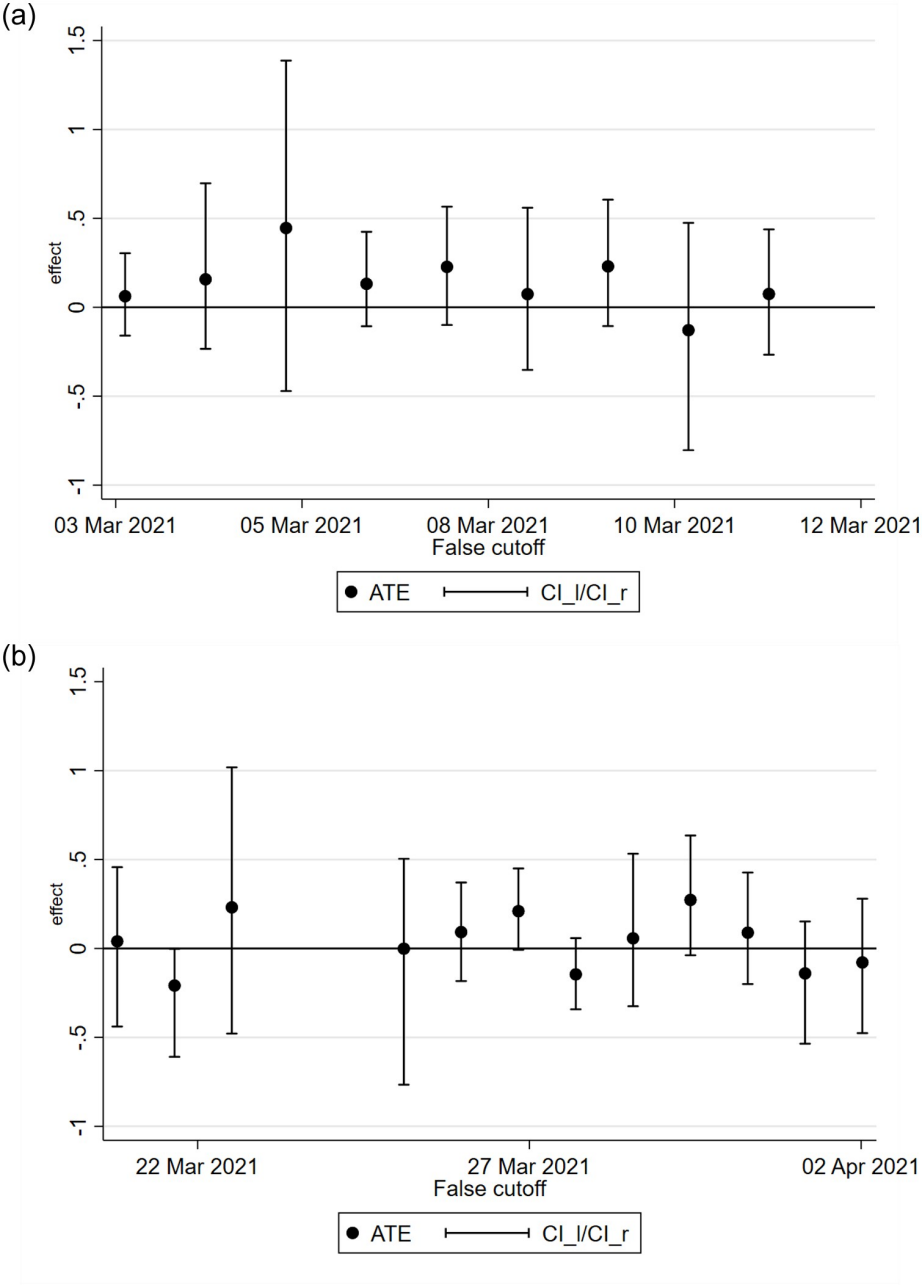
Placebo tests. *Notes*: Placebo tests point estimates and confidence intervals. The dependent binary variable is equal to 1 if the individual answers that they are willing to get vaccinated. In these placebo tests, *z* is the running variable on the time of survey completion, with a cut-off at 17:00 for each day to the left (graph (a)) or the right (graph (b)) of the true discontinuity (17:00 on the 18th of March). The missing point estimates are due to the low number of observations around some false cut-off points, which prevents the implementation of the RDD estimator. We follow Cattaneo14 with the following options: triangular kernel; variance-covariance matrix estimated using the heteroskedasticity-robust nearest-neighbour variance estimator and local linear polynomial regression based on the MSE-optimal bandwidth selector for each side of the cut-off.

Still, we conduct several validation tests to ensure the validity of our results. First, we implement standard density tests proposed in the literature [16] to check for the existence of mass points on the number of respondents around the cutoff, which may be a sign of manipulation. The density test of [17] confirms that this is not the case, with a robust bias-corrected p-value of 0.962 (see Fig 3). Second, we show that the observable composition of the respondents does not change at the cutoff by obtaining RDD estimates for our set of covariates *X* (see Table 4). Third, we implement a battery of placebo tests for each side by imposing a false cutoff on each day at 17:00. As shown in Fig 4, the estimates are never statistically significant. Fourth, we test the robustness of our results to the definition of our running variable *z* by using the time at which respondents *started* the survey. Table 5 shows that this definition of the running variable delivers even larger point estimates, though still within the confidence interval of our benchmark results.

**Table 4:** Effect on covariates.

VARIABLES	(1)	(2)	(3)	(4)	(5)	(6)	(7)	(8)	(9)	(10)	(11)
	Woman	Single	Age≥50	COVID dangerous	Lux. nationality	Graduate	Employed	High income	Daily TV	Low trust in gov.	Low trust in science
Effect at cut-off	0.230	-0.056	-0.019	0.140	0.212	0.166	-0.115	0.147	0.022	0.028	-0.131
Robust *p*-value	0.148	0.576	0.860	0.320	0.232	0.289	0.307	0.392	0.797	0.995	0.373
Robust 95% CI	[-0.09;0.60]	[-0.37;0.20]	[-0.44;0.37]	[-0.13;0.40]	[-0.10;0.41]	[-0.18;0.62]	[-0.36;0.11]	[-0.18;0.47]	[-0.51;0.39]	[-0.39;0.39]	[-0.41;0.16]
Y right	0.684	0.175	0.268	0.566	0.691	0.509	0.785	0.473	0.557	0.604	0.513
Y left	0.454	0.232	0.288	0.426	0.479	0.343	0.900	0.326	0.535	0.576	0.644
BW loc. poly. [h]—left	12/03/21 04:45	11/03/21 02:06	12/03/21 00:31	12/03/21 15:51	15/03/21 05:38	15/03/21 06:22	11/03/21 10:13	12/03/21 11:38	14/03/21 14:51	14/03/21 03:36	10/03/21 20:10
BW loc. poly. [h]—right	27/03/21 04:48	26/03/21 19:09	23/03/21 14:39	27/03/21 12:20	27/03/21 21:19	27/03/21 04:35	26/03/21 06:53	28/03/21 12:43	27/03/21 09:56	24/03/21 04:38	27/03/21 06:35
BW bias [b]—left	09/03/21 04:22	07/03/21 15:07	09/03/21 06:07	09/03/21 12:31	12/03/21 16:22	12/03/21 01:17	07/03/21 21:25	09/03/21 21:59	12/03/21 00:29	10/03/21 19:48	08/03/21 00:40
BW bias [b]—right	02/04/21 14:13	01/04/21 19:39	29/03/21 02:58	03/04/21 02:34	03/04/21 16:39	02/04/21 15:56	31/03/21 11:28	05/04/21 19:38	03/04/21 03:20	29/03/21 13:22	02/04/21 11:45
Order loc. poly. [p]	1	1	1	1	1	1	1	1	1	1	1
Order bias [q]	2	2	2	2	2	2	2	2	2	2	2
Covariates	No	No	No	No	No	No	No	No	No	No	No
N	696	696	690	690	696	696	696	695	691	695	692
Eff. N estimate [h]	208	200	142	213	211	194	181	234	198	132	214
Eff. N bias [b]	374	402	276	375	348	345	365	375	347	264	397

*Notes*: RDD estimates on the covariates as defined in Section 2. *z* is the running variable on the time of survey completion with a cutoff on the 18th of March. We followed Cattaneo14 with the following options: triangular kernel; variance-covariance matrix estimated using the heteroskedasticity-robust nearest-neighbour variance estimator and local linear polynomial regression based on the MSE-optimal bandwidth selector for each side of the cutoff. The table shows the optimal bandwidth for each side of the cutoff for the estimate (h) and the bias (b).

* significant at 10% level,

** significant at 5% level,

*** significant at 1% level.

**Table 5:** RDD estimates using time at the beginning of the survey as running variable.

	(1)	(2)	(3)	(4)
Effect at cut-off	0.603***	0.644***	0.610***	0.613***
Robust *p*-value	0.001	0.001	0.000	0.000
Robust 95% CI	[0.275; 1.050]	[0.293; 1.101]	[0.322; 0.936]	[0.302; 1.032]
Y right	0.872	0.862	0.862	0.869
Y left	0.269	0.218	0.232	0.111
BW loc. poly. [h]—left	14/03/21 13:30	11/03/21 14:46	15/03/21 02:11	14/03/21 07:41
BW loc. poly. [h]—right	23/03/21 23:58	31/03/21 13:39	23/03/21 19:45	30/03/21 03:47
BW bias [b]—left	11/03/21 11:49	09/03/21 19:31	13/03/21 06:16	12/03/21 13:18
BW bias [b]—right	29/03/21 12:45	04/04/21 03:06	28/03/21 23:26	31/03/21 14:05
Order loc. poly. [p]	1	2	1	2
Order bias [q]	2	3	2	3
Covariates	No	No	Yes	Yes
N	696	696	673	673
Eff. N estimate [h]	124	291	119	235
Eff. N bias [b]	244	355	222	277

*Notes*: The dependent binary variable is equal to 1 if the individual answers that they are willing to get vaccinated. *z* is the running variable on the starting time of the survey, with a cut-off at 17:00 on the 18th of March. We follow [16] with the following options: triangular kernel; variance–covariance matrix estimated using the heteroskedasticity-robust nearest-neighbour variance estimator. Different models: (1) local linear polynomial regression based on the MSE-optimal bandwidth selector for each side of the cut-off, (2) local quadratic polynomial, (3) local linear polynomial regression adding covariates, (4) local quadratic polynomial adding covariates. The table shows the optimal bandwidth for each side of the cut-off for the estimate (h) and the bias (b).

* significant at the 10% level,

** significant at the 5% level,

*** significant at the 1% level.

Finally, we create two alternative dependent variables. The information about the willingness to be vaccinated was collected via the following question: Do you intend to get vaccinated against COVID-19? Yes, absolutely (1)—Probably yes (2)—Probably not (3)—No (4). From this question our main outcome variable was based on a dummy variable taking a value of 1 if respondents answered (1) or (2), and a value of 0 otherwise. We therefore check the sensitivity of our results to different versions of the dependent variable by either opposing the definite ‘No’ (4) to the other answers, or the definite ‘Yes’ (1) to the other answers. We show in Table 6 that the results are robust to the first alternative formulation of the dependent variable, suggesting that the reinstatement led some individuals who definitely did not want to be vaccinated to reconsider their refusal. In contrast, opposing the ‘Yes, absolutely’ (1) to all other possible responses does not yield conclusive results (see Table 7). This is sensible since it is less likely that individuals who were already mildly in favour of vaccination would become absolutely convinced by the reinstatement.

**Table 6:** RDD estimates with a different outcome definition: Y = 1 if ‘Yes, absolutely’, ‘Probably yes’, or ‘Probably no’, Y = 0 if ‘Definitely no’.

	(1)	(2)	(3)	(4)
Effect at cut-off	0.432***	0.577***	0.368***	0.453M***
Robust *p*-value	0.005	0.002	0.001	0.003
Robust 95% CI	[0.148; 0.833]	[0.247; 1.050]	[0.160; 0.669]	[0.174; 0.870]
Y right	0.957	0.968	0.953	0.882
Y left	0.525	0.391	0.575	0.322
BW loc. poly. [h]—left	14/03/21 23:31	13/03/21 09:35	13/03/21 15:55	14/03/21 03:41
BW loc. poly. [h]—right	24/03/21 08:58	29/03/21 13:51	25/03/21 02:46	01/04/21 19:00
BW bias [b]—left	11/03/21 23:30	10/03/21 23:57	09/03/21 16:23	11/03/21 22:31
BW bias [b]—right	30/03/21 16:06	02/04/21 17:41	30/03/21 23:25	07/04/21 06:02
Order loc. poly. [p]	1	2	1	2
Order bias [q]	2	3	2	3
Covariates	No	No	Yes	Yes
N	696	696	673	673
Eff. N estimate [h]	130	252	129	303
Eff. N bias [b]	279	351	303	341

*Notes*: The dependent binary variable is equal to 1 if the individual answers that they may get vaccinated (‘Yes, absolutely’, ‘Probably yes’, or ‘Probably no’) and 0 otherwise. *z* is the running variable on the ending time of the survey, with a cut-off at 17:00 on the 18th of March. We follow [16] with the following options: triangular kernel; variance–covariance matrix estimated using the heteroskedasticity-robust nearest-neighbour variance estimator. Different models: (1) local linear polynomial regression based on the MSE-optimal bandwidth selector for each side of the cut-off, (2) local quadratic polynomial, (3) local linear polynomial regression adding covariates, (4) local quadratic polynomial adding covariates. The table shows the optimal bandwidth for each side of the cut-off for the estimate (h) and the bias (b).

* significant at the 10% level,

** significant at the 5% level,

*** significant at the 1% level.

**Table 7:** RDD estimates with a different outcome definition: Y = 1 if ‘Yes, absolutely‘, else Y = 0.

	(1)	(2)	(3)	(4)
Effect at cut-off	0.257*	0.301	0.104	0.120
Robust *p*-value	0.093	0.169	0.354	0.552
Robust 95% CI	[-0.045; 0.593]	[-0.119; 0.676]	[-0.138; 0.386]	[-0.266; 0.499]
Y right	0.501	0.382	0.514	0.434
Y left	0.244	0.081	0.251	0.190
BW loc. poly. [h]—left	14/03/21 05:23	14/03/21 04:57	14/03/21 09:11	12/03/21 05:04
BW loc. poly. [h]—right	26/03/21 14:36	29/03/21 04:42	27/03/21 06:37	30/03/21 14:30
BW bias [b]—left	11/03/21 07:31	11/03/21 18:13	12/03/21 01:57	09/03/21 19:59
BW bias [b]—right	01/04/21 22:45	01/04/21 14:42	02/04/21 16:27	02/04/21 07:27
Order loc. poly. [p]	1	2	1	2
Order bias [q]	2	3	2	3
Covariates	No	No	Yes	Yes
N	696	696	673	673
Eff. N estimate [h]	178	238	189	263
Eff. N bias [b]	340	326	329	344

*Notes*: The dependent binary variable is equal to 1 if the individual answers that they are willing to get vaccinated with certainty and 0 otherwise. *z* is the running variable on the ending time of the survey, with a cut-off at 17:00 on the 18th of March. We follow [16] with the following options: triangular kernel; variance–covariance matrix estimated using the heteroskedasticity-robust nearest-neighbour variance estimator. Different models: (1) local linear polynomial regression based on the MSE-optimal bandwidth selector for each side of the cut-off, (2) local quadratic polynomial, (3) local linear polynomial regression adding covariates, (4) local quadratic polynomial adding covariates. The table shows the optimal bandwidth for each side of the cut-off for the estimate (h) and the bias (b).

* significant at the 10% level,

** significant at the 5% level,

*** significant at the 1% level.

To conclude, our validation and robustness tests confirm the reliability of our estimates in representing the causal effect of the EMA statement of the 18th of March.

## 3 Discussion

**Fig 5:**
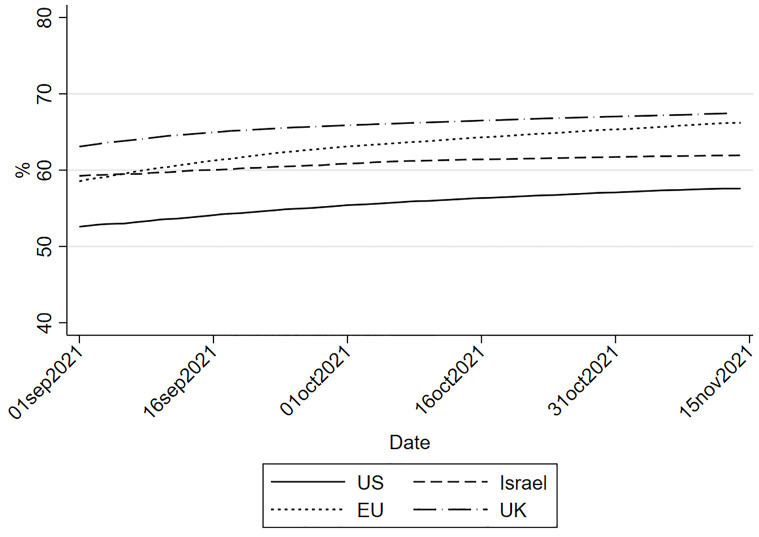
Evolution of the vaccination rates (full vaccinations) in the EU, the UK, Israel and the US. *Notes*: Source: https://ourworldindata.org/covid-vaccinations.

The global vaccination campaign against COVID-19 is one of the most crucial challenges in recent history, and vaccine hesitancy is arguably the most important factor threatening its success. The vaccination rates in the most proactive countries are indeed struggling to reach sufficient levels for the acquisition of herd immunity (see Fig 5). Various attempts are being made to convince vaccine-hesitant individuals to reconsider their position through ‘carrot and stick’ policies. Due to the potential emergence of more aggressive variants and of other viruses in the future, the battle against vaccine hesitancy remains a pressing issue.

Most of the research on public policies aiming to address vaccine hesitancy has focused on targeted interventions [2]. Recently in Europe, public actions fuelled hesitancy when 18 European governments suspended Vaxvevria following news reports of rare blood clots. Our data confirm that in the days that followed these events, intentions to get vaccinated severely declined.

In this paper, we study whether the communication of a supranational drug regulator, the EMA, and the coordinated vaccine reinstatement by 15 governments could restore intentions to get vaccinated to previous levels. While the intention to get vaccinated was at its lowest in the days preceding the EMA statement, we find that the vaccine’s reinstatement led to an increase in the intention to get vaccinated of about 50 percentage points.

Interestingly, our finding that the endorsement of the official regulator followed by a coordinated action allowed to rebuild confidence had been hypothesized by [19]. Considering the investments and stakes involved in this vaccination campaign, this result is particularly important. Moreover, this result has wider implications as the lack of coordination observed at the European level is susceptible to occur at more local levels of governance. Our results establish that maintaining a common vision and centralized approach is essential to reaching herd immunity at a global level.

Regarding the determinants of vaccination intent, We find that the willingness to get vaccinated is lower among people with a lower educational attainment, among individuals below the age of 50, and among individuals who do not perceive COVID-19 to be dangerous. Lack of trust in science and in government action also correlate negatively with the willingness to get vaccinated, whereas frequent traditional media consumption (TV) is positively correlated with vaccination intent.

**Fig 6:**
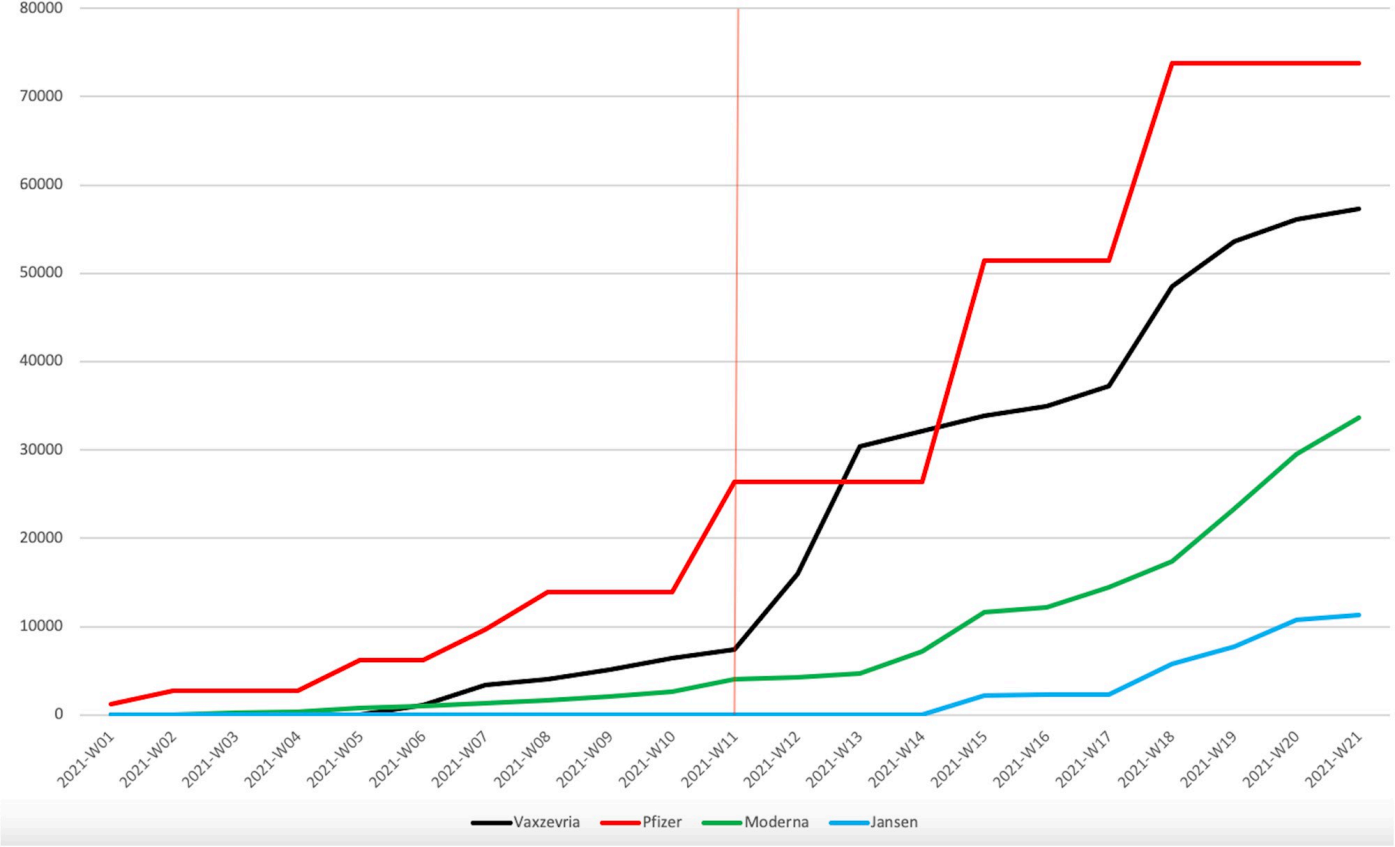
Cumulative number of doses administered in Luxembourg, by manufacturer. *Notes*: Source: https://vaccinetracker.ecdc.europa.eu.

Finally, we acknowledge that a reported intention to get vaccinated may differ from individuals’ actual behaviour. While our results identify a clear break between the pre- and post-EMA declaration periods in terms of vaccination intentions, this finding appears to also apply to actual vaccinations in Luxembourg. Fig 6 indeed shows that the total number of Vaxzevria injections in Luxembourg had stalled until week 11 (March 15 to March 21, 2021), and had increased drastically after week 11.

**Fig 7:**
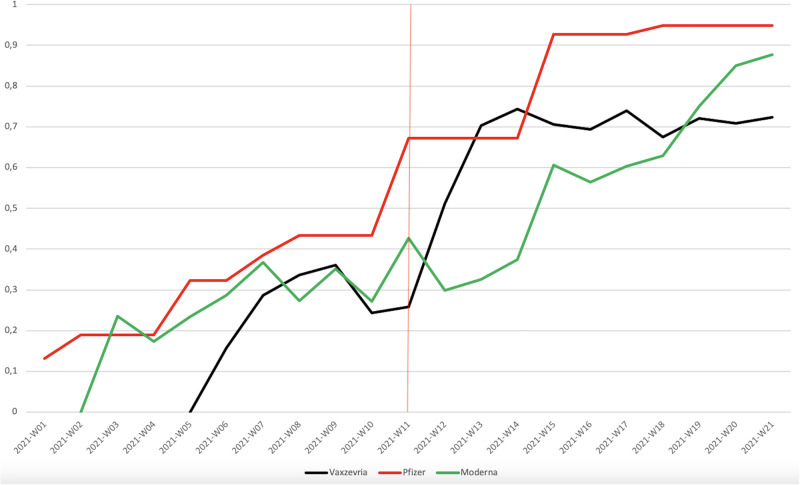
Proportion of administered doses in Luxembourg, by manufacturer. *Notes*: Source: https://vaccinetracker.ecdc.europa.eu.

We conclude the paper by discussing the evolution of vaccinations from other manufacturers relative to Vaxzevria during this period. The main alternatives were Pfizer-BioNTech and Moderna, and Fig 6 suggests that they were not subject to a break around week 11. Instead, injections of these two vaccines appeared to grow rather steadily over the studied period. One aspect that deserves attention here is the potential role played by capacity constraints and the government’s distribution strategy of the various vaccines. To account for this, Fig 7 presents the proportion of administered doses relative to the cumulative number of vaccine doses delivered to Luxembourg. This measure provides an indication of the government’s capacity -conditional on the population’s willingness- to distribute each vaccine relative to its available stocks. Initially, these proportions were low for all vaccines since the vaccination campaign was in its early phase. In the following weeks, these proportions followed a positive trend, with the exception of weeks 9 and 10, which correspond to the period in which blood clots were reported in the press. This decrease was the strongest for Vaxzevria, although Moderna also slightly stalled. Conversely, Pfizer stocks were widely used during this period. After week 11, the administered proportion of Vaxzevria increased drastically, in line with our finding that confidence was restored. At the end of the period, the usage proportions were similar across all vaccines.

## Supporting information

S1 Data (ZIP)
